# The Menstrual Health Manager (MHM): A Resource to Reduce Discrepancies Between Science and Practice in Sport and Exercise

**DOI:** 10.1007/s40279-024-02061-w

**Published:** 2024-06-21

**Authors:** Claire E. Badenhorst

**Affiliations:** School of Sport, Exercise and Nutrition, Massey University, Auckland, New Zealand

## Abstract

Inadequate research on female health and performance; the complexity of the research; low menstrual health literacy of athletes, coaches, and support staff; and ethical and cultural sensitivities are all recognized as barriers to effective health monitoring for females in sports. Frameworks have been developed for academics to follow to help improve the quality of female-specific research. However, a similar resource that enables correct terminology, and use of health monitoring techniques has not been provided for sporting organizations, coaches, support staff or athletes. Therefore, this critical commentary presents a new resource, the Menstrual Health Manager. This resource may be used to determine the level of menstrual health monitoring detail that may be used by organisations, coaches or athletes, and specifies what reproductive health details the data will provide. This resource aims to provide organizations and coaches with a means of understanding the data that inform their decisions for female athletes. Utilization of this resource may aid in the consistent use of terminology and methods for female-specific health monitoring in both sports and research.

## Key Points

**Table:** 

Inadequate research, low menstrual health literacy, and lack of educational resources and training are barriers that prevent effective menstrual health monitoring for females, for both active individuals and elite athletes.
The development of educational resources that use correct terminology (in lay language) is required for athletes, coaches and support staff. This strategy could help improve appropriate and effective menstrual cycle health monitoring for females in sport and exercise environments.
A decision tree has been constructed for coaches, sporting organizations and athletes to inform decision-making on what menstrual health monitoring methods could be used, while also ensuring that there is some understanding/learning of what information will be provided.

## Introduction

In 2023, the FIFA Women’s World Cup broke attendance records, drawing the largest crowds and ticket sales for a female-specific sporting event [1]. The year preceding this record-breaking World Cup event, the Tokyo Olympic Games demonstrated near parity in participation by male and female athletes, with Britain, Canada, China and Australia sending teams that were female-dominant [2]. However, the substantial growth in female sport participation has yet to be met with equality or equity in media coverage, salaries, athlete support (e.g., maternity leave, childcare support, mental and physical health), female leadership, female coaches, female-specific data and evidence-informed recommendations. As a result, in more recent times, an increase in sporting organization scrutiny by both athletes and media [3–5] has been observed. Organizations are criticized for their lack of female welfare and support, and subsequently calls to action have moved for improvements in policies and education from grassroots through to elite female athletes [5]. However, when turning to the research sector to inform education, performance and training recommendations, these organizations and individuals will find limited evidence [6].

To consolidate the available research on “How does the menstrual cycle affect exercise, sports performance, health, and well-being”, numerous audits of the available literature have been completed. These audits have investigated the extent of cis-female representation in the participant pool in sport medicine and exercise science journals [7], nutrition studies [8], research methodology [9, 10], menstrual cycle symptoms [11] and sport/exercise performance [12, 13]. Subsequently, these audits demonstrate how the issues of small sample sizes, the low quality in study design, and heterogeneity between studies and individuals have cumulatively contributed to a substantial cis-female data gap in sport and exercise science. As such, most sporting organizations have access to countless reviews that state that there is limited research evidence to inform adjustment of training, recovery or nutrition to the physiology of individuals who menstruate [8, 12, 13].

The data gap in cis-female research are also contributing to the consistent reports of low menstrual health literacy within sports [14]. Inadequate menstrual health literacy or menstrual health education will adversely influence the validity of the data provided by the individual on their menstrual health [15]. In addition, the reliability of menstrual health data may be questioned, especially when coaches and sporting organizations are faced with data-reporting compliance issues. For example, following the initial implementation of menstrual health data tracking, there may be high compliance by athletes due to its novelty. However, without coaches and support staff providing clear objectives on why data are being collected, there is a risk that once there is an increase in self-awareness or survey fatigue the consistency and reliability of data reporting may be reduced [15]. Of note, in the absence of athletes understanding why menstrual health data are being collected, the reporting of this health data may raise ethical concerns for athletes about how the data will be used by coaches and the organization [16]. Subsequently, counterarguments to the praise of menstrual health data collection and awareness within sports include misinterpretation of data, incorrect decisions on training, and performance, potential loss of privacy of the athlete, and disrespect for cultural sensitivities on the topic [16].

There is no argument that the menstrual cycle is a marker of female health. However, in the absence of high-quality research data, effective use of in-field data is likely to only occur once key issues pertaining to menstrual health literacy for athletes, coaches and support staff are addressed. Inadequate menstrual cycle and/or hormonal contraception knowledge of coaches has been suggested as a primary barrier to athlete communication, regardless of whether athletes rate their athlete-coach relationship favourably. Discussions with female coaches may occur more readily, but may still be limited to the coach’s education or influenced by the coach’s personal experiences/biases with their cycle. Exacerbated by the lack of formal education/discussions on the menstrual cycle and inadequacy of available research, any application of menstrual cycle tracking, monitoring or adjustment of training may be perceived as difficult, especially in the context of elite sports [17]. Therefore, education and resources that enable athletes, coaches and support staff to understand what they can monitor with regard to the menstrual cycle are required. This initial process may be beneficial to sporting organizations that wish to implement menstrual health discussions and health data collection in a culturally and ethically appropriate manner.

## Basis for Resource Development

In the interim, standardizing menstrual health terminology and methodology by organizations, coaches and athletes should be considered a priority. Previously, academic experts in the field of sport and exercise have provided guidelines and a framework for current and future academics to consider when designing cis-female-specific in-lab research projects [10]. The provision of this framework has sought to remove the barriers that will enable high-quality research projects that are then able to provide evidence-based guidelines for female exercisers, their coaches, and support staff. Specifically, this work provided definitions of the female reproductive system and the diversity that may need to be considered throughout the lifespan both within and between cis-females. However, it is recognized that this paper was designed to improve research quality, and as such meets the needs of its primary target audience, academics and researchers in medicine, exercise and sport science. Therefore, the usability of this paper for applied, in-field support and education is limited.

Previous research acknowledges that even if menstrual health information is acquired by coaches, the complexity of the information remains a barrier to its usability (e.g., communication, understanding and awareness) [17]. Training management and physical performance are key considerations for coaches who desire a greater understanding of the physiological nuances that occur when working with female athletes [18]. To date, there is insufficient evidence available to suggest individualized training based on menstrual cycle phases or characteristics [13]. There may, however, be a consideration for individualized recovery and support provided to female exercisers/athletes that considers their unique physiology [18]. However, this may only be achieved with both coaches and athletes being aware and having transparent agreements on (1) what health data are being provided, (2) how these data may affect the discussions and management of the menstrual cycle, and (3) what data should be considered, collected and reported to ensure the individual’s privacy is maintained. As such, the following resource has been developed for cis-female athletes/exercisers, coaches and support staff to inform their decisions on menstrual health monitoring. The resource should enable any implemented health monitoring processes to remain in alignment with guidelines provided by academic experts in female-specific exercise and sport science.

## Menstrual Health Manager (MHM)

**Fig. 1:**
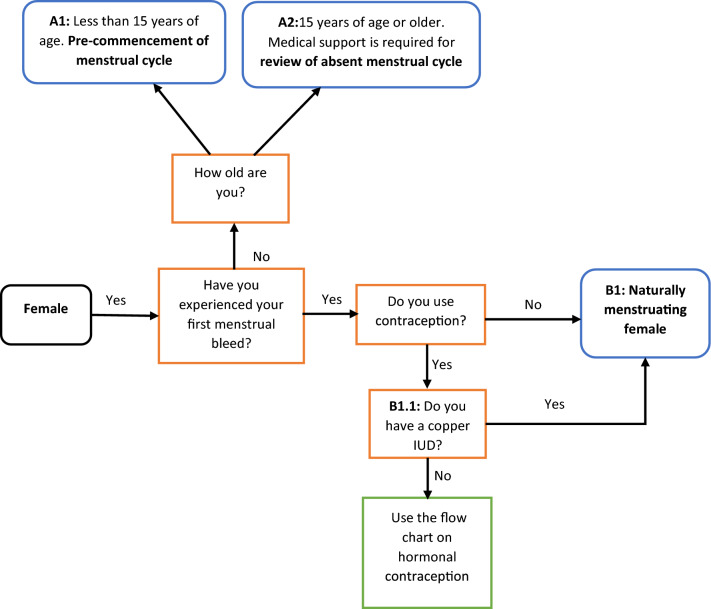
A basic decision tree for determining menstrual cycle status. Commencing at the black root node ‘female’, simple binary questions (orange) can be completed to move progressively until a leaf (blue) node is reached. If responses to binary questions land on the green leaf node, then the individual using the resource should progress to Fig. 4. *IUD* intrauterine device

**Fig. 2:**
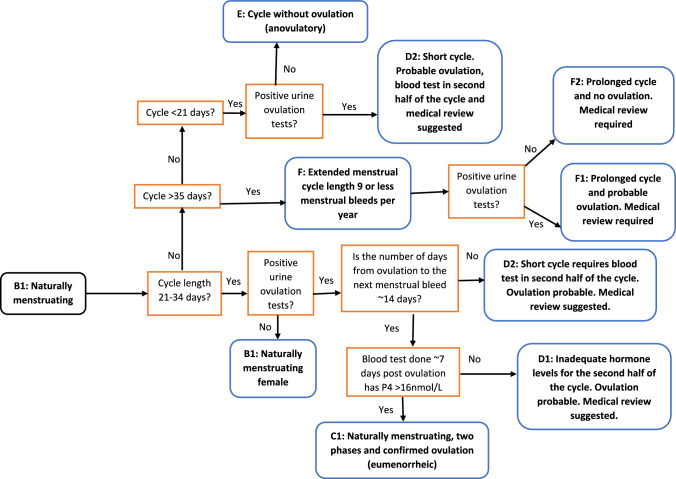
An extension of Fig. 1 that can be used to determine the menstrual cycle status of a naturally menstruating female (black root node). Answering the menstrual cycle monitoring binary questions (orange) will progressively move the individual towards a leaf (blue), helping to establish the menstrual cycle status of the individual in each cycle. Detailed descriptions of each leaf (blue) are provided in Table 1. The menstrual cycle monitoring techniques and questions (orange) that are required to complete this decision tree are outlined in Table 2. Of note, ovulation in the decision tree has been stated as probable if a urinary ovulation test (LH surge) has been completed and is positive. To confirm ovulation a progesterone (P4) blood test will need to be completed 7–9 days after the urinary ovulation test. *LH* luteinizing hormone

**Fig. 3:**
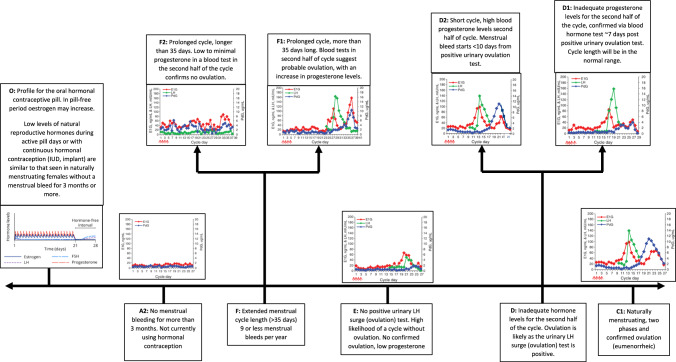
An overview of the continuum of female hormonal profiles, adapted from Allaway et al. [43], that correspond to each leaf in the naturally menstruating female decision trees (Figs. 1 and 2). After the individual has worked their way through the decision trees to identify the appropriate leaf and hence the menstrual cycle status of the individual, the assigned code on each leaf can be linked to the image and lay summary provided here or to a written description provided in Table 1. *E1G* urine metabolite of estrogen, estrone-3-glucuronide, *PdG* urine metabolite of progesterone, pregnanediol glucuronide, *LH* luteinizing hormone, *FSH* follicle-stimulating hormone

To enable simple and informed decision-making on what menstrual health monitoring methods could be used, while also ensuring that there is some understanding/learning of what information will be provided, decision trees for menstrual cycle status have been developed (Figs. 1, 2, 3). Decision trees have been used in medical and healthcare settings to provide a support system that enables reliable and effective decisions to be made by practitioners [19, 20]. Decision trees provide a means of classification based on a series of questions on the features or characteristics associated with data [20]. The tree commences at the root node, and in this instance, the root node of ‘female’. The decision tree provides a series of questions, and each of these questions is contained within a node. In a simple binary format, each question of the decision tree has a simple yes or no response, with each possible answer directing the user to a specified child node [20].

Within this paper and the decision trees, the term ‘female’ was used to describe individuals designated with the biological sex characteristics that would enable menstruation to occur [21]. Historically, ‘female’ and ‘women’ post-puberty have both been used in the research literature to describe individuals who menstruate. However, it is acknowledged that the term ‘women’ may be used to describe the individual’s gender, with gender recognized as a social construct that depicts the roles and behaviors of individuals [21]. The decision tree has been designed for cis-gender females based on the presence of ovarian steroid concentrations. However, both sex and gender are not binary, and this may need to be considered when using this resource.

There are two outcomes when using the decision tree as a means of understanding what methods can be used to monitor menstrual health that athletes, coaches or support staff may consider. Firstly, users may select to work from the root node through a series of questions to a node that has no further questions, i.e. a leaf. To ensure adequate understanding and learning when using the resource, an expanded definition of each leaf of Figs. 1 and 2 is provided in Table 1, while a lay summary and image of the hormonal profile (endogenous estrogen and progesterone levels) associated with each leaf are provided in Fig. 3. For each leaf, a code has been provided to allow the user to find the appropriate menstrual cycle profile (Fig. 3) or description (Table 1). Users may then select to implement the in-depth menstrual health monitoring protocol that enables them to reach the relevant leaves. Details on what menstrual health monitoring tools or methods may be used to help respond to each of the binary questions within Fig. 2 are provided in Table 2. In Fig. 2, to ensure alignment with the recent IOC-REDs Clinical Assessment Tool [22], the suggestion of medical support to review the menstrual health status or required medical review of the identified menstrual health status has been provided.

**Table 1:** Leaf description, non-clinical and clinical definitions, and associated characteristics for naturally menstruating females

Leaf description	Clinical description	Non-clinical description	Characteristics
	Puberty	Puberty	Onset/start of menstrual bleeding in females
	Menarche	First period	First menstrual bleed
A1: Less than 15 years of age. Pre-commencement of the menstrual cycle	Primary Amenorrhea	No menstrual bleed by the age of 15 years	Lack of or absence of first menstrual bleed by the age of 15 years, despite secondary sex characteristics being present. Or the absence of the first menstrual bleed by the age of 14 years and no secondary sex characteristic present [10]
A2: 15 years of age or older. Medical support is required for review of absent menstrual cycle	Functional hypothalamic amenorrhea (FHA) or secondary amenorrhea​	Older than 15 years of age. Menstrual bleeding has stopped for 3 months or more	Absence of menstrual bleeding despite the female having experienced menarche, which may result from various stressors (psychological, nutritional, physical). All other medical causes of amenorrhea must be excluded prior to diagnosis. No medication or contraception is currently being used which may be the cause of menstruation absence [10]
B1: Naturally, menstruating female	Naturally menstruating female​	Have a regular menstrual bleed and not using hormonal contraception	Individuals will have regular menstrual bleeds, typically occurring within a 21–35 day cycle, and they will have more than 9 menstrual bleeds a year. Unable to classify them as eumenorrheic, luteal phase defect, or anovulatory as urinary ovulation test (luteinizing hormone (LH) surge) and blood tests have not been completed. Ovulation and adequate blood concentrations of the progesterone have not been confirmed [43, 44]
C1: Naturally menstruating, two phases and confirmed ovulation (eumenorrheic)	Eumenorrheic biphasic cycle​	Naturally menstruating, has confirmed ovulation, and confirmed hormone concentrations in the first and second half of the cycle	Individuals with regular menstrual bleeds that occur within a 21–35 day cycle. Positive urine ovulation tests confirm LH surge. The luteal phase is ~ 14 days in length and the mid-luteal phase blood progesterone levels are > 16 nmol/L, confirming ovulation and two distinct phases of the reproductive hormone profile [10, 43, 44]
D1: Inadequate hormone levels for the second half of the cycle. Medical review required. Confirmed ovulation OR D2: Short cycle. Probable ovulation, blood test in second half of the cycle and medical review required	Luteal phase defect​	Naturally menstruating, disrupted second half of cycle, specifically short or low progesterone hormone concentrations	Individuals who have 21–35 day cycles and experience 9 or more cycles a year and have positive urinary ovulation test results. Mid-luteal blood progesterone levels are less than 16 nmol/L OR the onset of subsequent menstrual bleed occurs < 14 days after a positive urinary ovulation test (LH surge occurred). The presence of progesterone, even at low levels, confirms ovulation has occurred [44]. Often missed as menstrual cycle length does not change and is only detected if urinary ovulation tests and mid-luteal blood tests are completed
E: Cycle without ovulation. No positive urine ovulation tests, no positive LH surge detected	Anovulatory​	Naturally menstruating females that when tested do not have a positive ovulation test (urine or blood)	Individuals may experience regular menstrual bleeds (every 21–35 days), and experience 9 or more cycles a year but urinary ovulation tests are negative (no LH surge detected). Lack of ovulation in this cycle is confirmed with low mid-luteal phase blood progesterone levels [44]
F: Prolonged cycle with or without ovulation. Medical review required	Oligomenorrheic ​	Extended menstrual cycle length 9 or less menstrual bleeds per year	Individuals whose menstrual bleeds occur less regularly and may occur every 35 days or more. They may have less than 9 menstrual bleeds in a year [10] These cycles can be both anovulatory or ovulatory, but this classification requires confirmation of ovulation through positive urinary ovulation (LH surge) testing and phase-specific blood tests [44]

*LH* luteinizing hormone, *FHA* functional hypothalamic amenorrhea, *Nmol/L* nanomol/L

**Table 2:** Menstrual cycle monitoring variables, their usability, and considerations for females

Monitoring or testing option	Group	Characteristics	Notes	Frequency of use
Menstrual cycle duration	Naturally menstruating females	Tracking the number of days can be completed via app, diary, or calendar method. Can provide details on the regularity of the menstrual bleeds and can help determine if they are oligomenorrheic (> 35 days) or have a potential luteal phase defect (< 21 days) [43]	Does not confirm if the individual has ovulated and therefore cannot confirm if they are eumenorrheic, ovulatory or anovulatory. It cannot be used by OCP users	Daily
Menstrual cycle symptoms	Naturally menstruating and hormonal contraceptive users [45]	Individuals will not only present with menstrual-related symptoms during their menstrual bleed or in the 3–5 days preceding the bleed. While these days within the cycle are recognized for the augmentation of an individual’s symptoms, there are likely to be subtle variations in symptoms throughout the cycle that impact the perception of health, well-being, and performance [46]	The individual’s data should aim to be collected every day at roughly the same time of day. Try to avoid retrospective analysis, as this may be biased towards the extremes in menstrual symptoms that occur prior to and during menstrual bleeding. Variations in symptoms may occur between cycles and between individuals [45]	Daily
Urinary ovulation test	Naturally menstruating females	Urinary ovulation test for the luteinizing hormone (LH) surge. An LH surge typically occurs mid-cycle (around day 14 in a standard 28-day cycle) in response to high estrogen levels causing ovulation in naturally menstruating females [43, 44]. Testing is readily available for females and can be completed at home	Testing is advised to start on day 8 of the cycle and continue until a positive test result is obtained [43]. Tests can be completed in the morning or evening as long as they are consistently completed at the same time of day [47]	Per cycle ~ 6–12 days depending on individual cycle length
Basal body temperature	Naturally menstruating females and OCP users	Progesterone will increase basal body temperature in the second half of the menstrual cycle. If regularly completed, this measure can be indicative of ovulation and can help determine luteal phase length	Testing requires strict adherence to procedures and requires a morning measurement, prior to any movement or activity [48, 49]. Illness and poor sleep may impact the results and should be noted [50, 51]. Research has demonstrated that natural temperature changes may still be present in OCP users [52]. Note: adherence to the required procedures is required to ensure accurate results	Daily
Cervical mucus	Naturally menstruating females, hormonal IUD, and implant	Following ovulation and during the luteal phase when progesterone levels are high, cervical mucus will change from an egg white consistency to thick, dry and paste-like. In hormonal contraceptive IUD and implant individuals, thickening of cervical mucus is a noted effect of the low-dose synthetic progestin [53]	Can be used as a feature in menstrual cycle tracking and symptoms. However, this will require education on the changes and variations throughout the cycle [54]	Per cycle ~ 1–4 days
Blood tests	Naturally menstruating females	Blood tests in mid-luteal phase (7–9 days post positive urine ovulation test). The mid-luteal test can confirm if the individual is eumenorrheic (mid-luteal progesterone levels > 16 nmol/L) or has a luteal phase defect (< 16 nmol/L) [10]	To confirm ovulation only the mid-luteal phase blood test is required. Can not be used by hormonal contraceptive users as the provision of synthetic hormones prevents natural fluctuations in estrogen and progesterone [55]. Is invasive and requires medical appointment and may have a cost associated with it	Quarterly
Schedule of OCP use	OCP users	Individuals may not be taking the pill in a 21/7 cycle. They may choose to extend the time on the active pill (double or triple) [56]. Reasons for extended use may be medical (e.g., to reduce the impact of withdrawal symptoms) [57] or may be a method used to adjust the timing of withdrawal bleed and avoid occurrence during the competition schedule [58]	For extended use of the OCP beyond 3 repeats of the active pill, medical support, and advice may be considered [59]. Breakthrough bleeding may occur within initial cycles of continuous use. This may then subside to amenorrhea (absence of menstrual bleeding) [60]	Monthly or per OCP cycle
Brand and generation of OCP	OCP users	The concentration of the synthetic hormones will vary between brands and generations of OCP [35]. During the active pill ingestion period, synthetic doses of hormones may gradually increase. Different brands and doses may influence individuals positively or negatively [12]	Third generation of most OCPs are available and with each generation doses and types of synthetic hormones are adjusted. However, second-generation pills may still be prescribed to women	Quarterly, or when changed

*OCP* oral contraceptive pill, *LH* luteinizing hormone, *IUD* intrauterine device, *nmol/L* nanomol/L

Secondly, sporting organizations may use the decision trees to inform health monitoring processes. Thus, identify the amount of personal health data they may consider collecting, ensuring ethical and cultural sensitivities are respected. Specifically, it is acknowledged that there are ethnic and cultural differences in knowledge, practices and traditions concerning the menstrual cycle. The use of methodologies (e.g., blood sample collection, cervical mucus) that do not consider these diverse values of athletes could increase the risk of discrimination of individuals in an organization and result in inaccurate data due to the lack of engagement in implemented health monitoring [23]. In these instances, to ensure cultural safety is maintained, the users may work through the decision tree from the root node, selecting to stop at a more conservative node and not progress with any further questions to a leaf. Again, Table 2 may be used to help inform which menstrual health monitoring practices coaches and sporting organizations may use to inform health-specific decision-making with female athletes. For both circumstances, the author has provided the frequency with which menstrual health monitoring tools may be used to help inform decision-making and support provided to female athletes. These recommendations are based on the work previously completed by academic experts who have experience in female athlete health monitoring [24].

**Fig. 4:**
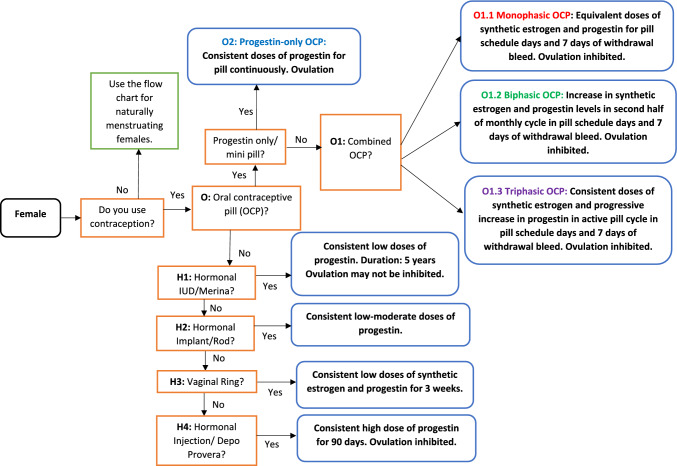
Basic decision tree for hormonal contraceptive users. Commencing at a root node (black), binary questions (orange) can be completed until a leaf node is reached (blue). Details of each leaf node are provided in Table 3 and can be used collectively to provide education on the different types of hormonal contraception available to females. Details on menstrual health monitoring techniques for hormonal contraceptive users are provided in Table 2. *OCP* oral contraceptive pill, *IUD* intrauterine device

In recognition that not all premenopausal females may be naturally menstruating, a decision tree for hormonal contraception, a common hormonal milieu of active females [25–27], has been provided (Fig. 4). Hormonal contraception is defined as the provision of exogenous reproductive hormones that suppress endogenous reproductive hormone levels and may inhibit ovulation for the duration of time that the hormonal contraception is used [12]. The exception is the hormonal intrauterine device (IUD), where localized secretions of progestins have been shown to not inhibit ovulation throughout the duration of use [28].

Insights from research have suggested that the most utilized form of hormonal contraception is the combined oral contraceptive pill [25, 27, 29–31]; note, however, that there are many alternatives that females may have been prescribed [32–34], or have selected based on individual preferences. Due to different types, and brands, as well as the duration of exogenous hormone dose [12, 35], variations in exogenous hormone concentrations may occur. Therefore, while the choice of hormonal contraception remains that of the individual, support staff or coaches may use the flow chart to understand the various types that females may use and the methods by which they use these exogenous hormones (e.g., schedule). The flow chart is not provided as a tool to adjust or advise on hormonal contraception use: rather it is provided as an educational resource to inform athletes, support staff and coaches of the different types of hormonal contraception that an individual may use. This resource may be used to understand how hormonal contraception forms differ from each other and to naturally menstruating females. Additional details of each type of hormonal contraception are provided in Table 3. Finally, what health monitoring approaches may or may not be used for females who choose to use hormonal contraceptives have been provided in Table 2.

**Table 3:** Contraception type, exogenous hormones, and associated characteristics for females using hormonal contraceptives (see Fig. 4)

Contraception type	Characteristics
O: Oral contraceptive pill (OCP)	Daily pills are ingested by the individual and contain low doses of synthetic hormones ethinylestradiol and progestin. Twenty-one pills are provided with synthetic hormones and seven pills that do not contain synthetic hormones (e.g., placebo or sugar pills). Daily ingestion prevents the development of follicles in the ovaries and the occurrence of ovulation. Natural estrogen and progesterone levels are suppressed and remain low throughout the time when taking the pill [12]
O1: Combined OCP	Oral contraceptive pills that contain low doses of both ethinylestradiol and progestin [61]
O1.1 Monophasic combined OCP	Oral contraceptive pills that contain low doses of both ethinylestradiol and progestin and the dose remains the same throughout the 21 days of pills [61]
O1.2 Biphasic combined OCP	Oral contraceptive pills that contain both ethinylestradiol and progestin. However, the dose of ethinylestradiol over 21 days will stay the same while the progestin dose will increase in the second half of the hormone pill cycle [61]
O1.3 Triphasic combined OCP	Oral contraceptive pills that contain both ethinylestradiol and progestin. Over the 21 days of the pill, ethinylestradiol doses will remain constant, but progestin levels will increase every 7 days [61]
O2: Progestin-only or ‘mini-pill’	Form of OCP that contains only low doses of progestin for 28 days, unless a 7-day pill-free period is completed by the individual [61]
H1: Hormonal IUD or Mirena	An IUD that is placed inside the uterus of the individual and contains slow-release progestin. Typically lasts for 5 years, and over time the dose of progestin will decrease. As a result, the suppression of ovulation will decrease, and ovulation may start to occur more readily in the later years of the IUD lifecycle [62, 63]
H2: Hormonal implant or rod	Two small rods are placed under the skin of the upper arm. It contains slow-release progestin and will typically last for 5 years. Ovulation is likely to be suppressed for the duration of use [62]
H3: Vaginal ring	A ring that is inserted into the vagina and contains low doses of synthetic hormones ethinylestradiol and progestin. Typically inserted for 3 out of 4 weeks, but can be worn continuously [62]
H4: Depo Provera or Injection or Depo	A contraceptive injection containing moderate doses of progestin, requires regular doses every 13 weeks [62]
Withdrawal bleed	During a 7-day pill-free period (placebo pills, hormone-free or sugar pills), individuals may experience a withdrawal bleed. This is not a menstrual bleed as they have not ovulated. Rather the bleed occurs as a result of synthetic hormones being metabolized/broken down. During these 7 days, natural estrogen levels may increase [12]
B1.1: Copper IUD	An IUD that is placed into the individual’s uterus and is 99% effective in preventing pregnancy. Depending on the type inserted, it can last for 3, 5 or 10 years. Contains copper (metal) and does not contain any synthetic hormones and does not stop ovulation. Females may still have regular menstrual bleeds every 21–35 days and have 9 or more menstrual bleeds a year and will be classified as naturally menstruating [64]

*OCP* oral contraceptive pill, *IUD* intrauterine device

The decision tree has primarily been made for cis-females who are classified as premenopausal, and future versions of the resource may incorporate decision trees for females at different life-cycle stages. However, in recognition of the various life-cycle stages that females may experience, Table 4 has highlighted some critical changes in female reproduction, detailing the basic physiological changes and subsequent presentations and considerations for health monitoring.

**Table 4:** Additional lifecycle phases of females, reproductive hormone characteristics, and considerations that influence health and exercise monitoring

Physiological stress	Characteristics	Presentations and considerations	Monitoring
Pregnancy	Change in hormonal concentrations [65–71] Elevated progesterone Rise in prolactin from week 10 of gestation Rise in relaxin in the first trimester and decline in the second trimester Increase in estrogen throughout the pregnancy, peaks weeks 35–40 Vasopressin levels remain low throughout pregnancy Oxytocin levels progressively increase and parallel progesterone and estrogen Total plasma cortisol levels increase to three times non-pregnant levels by the third trimester Increased levels of aldosterone In response to a glucose load, increased release of insulin and suppression of glucagon	Basal body temperature is likely to be elevated, in the early stages of pregnancy [72] Relaxin increases in the first trimester may be associated with laxity around muscles and joints [73] Increased breast tissue enlargement; therefore, comfort and support for breast tissue may need to be considered more regularly [74, 75] The detrimental effects of high levels of cortisol and aldosterone would appear to be mitigated by high progesterone levels [76] Exercise has been noted as an effective method to help regulate glucose and insulin levels throughout pregnancy [77]	Daily: Symptoms, wellness, and readiness, sleep Weekly: exercise programme (volume, mode and intensity) and breast support Weekly/bi-weekly: Nutrition
Post partum/breast feeding	Elevated levels of oxytocin and prolactin for breast-feeding individuals [78] The hormonal milieu of breast-feeding may inhibit ovulation; however, this is individual-dependent In non-lactating individuals, the typical time frame for ovulation to return postpartum is 6–13 weeks, but this varies significantly between females [79, 80]	Individual recovery rates postpartum will vary Medical support and review will be required depending on the delivery Large drops in progesterone and estrogen may impact mental well-being [81] Initial postpartum hormonal milieu may include high cortisol and relaxin [67] levels. The intensity, mode, and duration of activity choice will depend on the individual [82] High nutritional requirements for recovery and breastfeeding [83]	Daily: Wellness, fatigue, sleep, physical recovery Weekly/bi-weekly: Nutrition, exercise programme (volume, mode, and intensity) Per cycle: ovulation and reproductive hormones Quarterly: health biomarkers (e.g., iron status)
Peri-menopause	The transition phase between reproductive years and non-reproductive years Estrogen levels may be higher and demonstrate erratic peaks as the individual approaches’ menopause. This is a result of high levels of follicle-stimulating hormone Lower exposure to progesterone results from increased incidence of luteal phase defects (low progesterone concentration and duration of luteal phase) and anovulatory cycles [84]	Increased heaviness of menstruation, and incidence of heavy menstrual bleeding Shorter and more regular cycles, a result of a shortening luteal phase Later stages of transition, increased incidence of oligomenorrhea or skipping menstrual bleeds Increased breast tenderness Increased sleep disruptions The onset of night sweats, more frequently occurring pre-menstruation Increased incidence of migraines Increased or new premenstrual mood disruptions/changes Weight gain or changes noted in body composition Bone health is a priority, and should be supported through the provision of strength-based exercise [84] Individuals may choose to utilize hormone replacement therapy (HRT) [10, 85]	Daily: Symptoms, wellness, and readiness, sleep Weekly/bi-weekly: Nutrition and exercise programme (volume, mode and intensity) Quarterly: Breast support, health biomarkers. HRT impact review and update- individual dependent
Menopausal	Twelve months with no naturally occurring menstrual bleed Estrogen and progesterone levels are low [86]	No menstrual bleed Bone health is a priority Changes in body composition likely [85–87]	Daily: Wellness, and readiness, sleep Quarterly: Nutrition and exercise programme (volume, mode and intensity)

*HRT* hormone replacement therapy

In recognition of common menstrual cycle disorders that females, both athletes and active individuals, may present with, Table 5 again defines the disorder and considerations for health, well-being and exercise/training monitoring. These definitions and considerations have been based on previous research on each of the conditions [36–42]. Details on health monitoring have been provided to support organizations or staff working with athletes at these various life-cycle stages or with a menstrual cycle disorder, providing a conservative framework to consider when implementing female health monitoring in sport and exercise. The resources are not diagnostic tools. Rather, if support staff or coaches identify common symptoms of disorders as a result of the menstrual health monitoring process, then it is advised that they seek out medical support and consultation.

**Table 5:** Common menstrual cycle disorders and considerations for health and exercise monitoring

Menstrual cycle disorder	Definition and characteristics	Considerations	Monitoring
Endometriosis	A chronic disease that affects 1 in 9 females. Defined by growth and inflammation of endometrial tissue outside of the uterus Often treated with hormonal therapy (oral contraceptives or hormonal IUD) Pain medication may be frequently prescribed In severe cases surgery is required The time frame for diagnosis is ~ 7 years (long) [37]	Symptoms include, but are not limited to [88]: Chronic pelvic pain Dysmenorrhea (menstrual pain) Dyspareunia (painful sex) Dysuria (painful urination) Dyschezia (painful defecation) Mid-cycle bleeding GI issues including diarrhoea and constipation Infertility Myofascial pain Fatigue Personalised symptom management is recommended During intense symptom presentation, adjustment of exercise mode, intensity and duration is advised [89]	Daily: Menstrual symptoms, wellness, and readiness, sleep, fatigue Per cycle: pain, fatigue, dose and frequency of pain medication Quarterly: Nutrition and exercise programme
Polycystic ovarian syndrome	An ovarian disorder characterized by high androgen levels, ovulatory dysfunction, and polycystic ovaries Prevalence of 3–18% in premenopausal females [90] Increased incidence of oligomenorrhea Estrogen levels are likely to be in the normal range or high [91]	Increased risk of co-morbidities including [92, 93]: Type II diabetes Insulin resistance Obesity Infertility Cardiovascular and cerebrovascular complications High iron status Exercise is beneficial for weight management and reducing co-morbidity risk	Daily: Menstrual symptoms, wellness, and readiness, sleep, fatigue Per cycle: days and presence of menstrual bleeding Quarterly: Nutrition, body composition, health biometrics (including iron, inflammation, diabetic and CVD parameters)
Premenstrual syndrome, premenstrual dysphoric disorder and dysmenorrhea	Primary dysmenorrhea: recurrent lower abdominal/or pelvic pain during menstruation Premenstrual syndrome: presentation of at least one somatic and affective symptom 3–5 days before menstruation and 1–3 days during menstruation [94] Premenstrual dysphoric disorder: severe form of PMS with augmented affective symptoms [95]	Symptoms will be notably present 1–5 days before menstruation and 1–3 days during menstruation and include [94, 95]: Pain Abdominal bloating Breast tenderness Headache Swelling of extremities Anxiety Irritability, anger outburst Confusion Depression Social withdrawal	Daily: Menstrual symptoms, wellness, and readiness, sleep, fatigue Per cycle: days, and presence of menstrual bleeding
Heavy menstrual bleeding	Loss of more than 80 ml (1/3 cup/4.5 tablespoons) of menstrual blood during menstruation [96] Complaint of heavy cyclical menstrual bleeding for more than 7 cycles [97]	Increased risk of iron deficiency [98] Increased fatigue and declines in performance [36] Increased anxiety and stress during menstruation [38] Performance and attendance may be affected	Per cycle: presence of menstrual bleeding, menstrual symptoms, duration of menstrual bleeding within the cycle and rating of volume lost per day Daily: wellness, and readiness, sleep, fatigue Monthly: fitness and exercise performance measures Quarterly: health biometrics (specifically iron status)

*CVD* cardiovascular disease, *IUD* intrauterine device, *PMS* premenstrual syndrome

## Conclusion

The decision trees and tables in this paper have been developed to educate and assist athletes, sports organizations and coaches with a framework that will enable them to develop or review their menstrual health monitoring methods. They are not suggested or intended as resources that can be used as diagnostic tools. It is anticipated these resources will help determine the details of menstrual health monitoring that may be implemented, while also ensuring that an improved understanding of the menstrual cycle occurs while still acknowledging the ethical and cultural perspectives of the individual/s providing the data. As such, these resources are likely to provide options for improved female health and well-being support and communication within a sporting environment that have previously been identified as barriers for coaches and support staff.

## Data Availability

As this is a short review and commentary all data is in the figures and tables which are presented as part of the article. There is no additional data that requires an availability statement.
